# Liver Transplantation: Does Prolonged Storage Promote Non-Anastomatic Biliary Structures?

**DOI:** 10.1155/1996/48793

**Published:** 1996

**Authors:** D. Kahn

**Affiliations:** Organ Transplant Unit Department of Surgery University of Cape Town Medical School Observatory Cape Town 7925 South Africa

## Abstract

The occurrence of biliary stricutures in allografts following liver transplantation correlates with the duration of preservation time. The correlation between preservation time and biliary strictures suggests that anoxic or reperfusion injury of the bile duct epithelium causes stricture formation. However, the relative susceptibility of bile duct cells to anoxic or reoxygenation injury is unknown. Our aims were to determine the vulnerability of rat liver bile duct cells to anoxic and reoxygenation injury and to compare the results with hepatocytes. During anoxia, bile duct epithelial cells were significantly more resistant to cell killing than hepatocytes. Rates of cellular proteolysis were also 2.5-fold lower in bile duct cells than in hepatocytes during anoxia. In contrast to anoxia, reoxygenation of anoxic cells increased cell killing of bile duct cells but improved viability of hepatocytes. The rate of toxic oxygen species formation by bile duct cells was 5-fold greater than in hepatocytes during reoxygenation. In addition, basal levels of glutathione are lower in bile duct cells than in hepatocytes. These data suggests that bile duct cells are more susceptible to reoxygenation injury than to anoxia. These studies support the hypothesis that reoxygenation injury during liver preservation leads to bile duct injury during liver transplantation.


*HPB Surgery*, 1996, Vol. 9, pp. 113–120
Reprints available directly from the publisher
Photocopying permitted by license only

© 1996 OPA (Overseas Publishers Association)
Amsterdam B.V. Published in The Netherlands
by Harwood Academic Publishers GmbH
Printed in Malaysia

### HPB INTERNATIONAL

**EDITORIAL & ABSTRACTING SERVICE**

JOHN TERBLANCHE, EDITOR
Department of Surgery,
Medical School · Observatory 7925
Cape Town · South Africa
Telephone: 27-21-406-6232,
Telefax 27-21-448-6461
Email: JTER BLAN @UCTGSHI.UCT.AC.ZA.

### LIVER TRANSPLANTATION: DOES PROLONGED STORAGE PROMOTE NON-ANASTOMATIC BILIARY STRUCTURES?

### ABSTRACT

*Noack, K., Bronk, S.F., Kato, A. and Gores, G.J. (1993) The greater vulnerability of bile duct cells to reoxygenation injury than to anoxia. Transplantation 56: 495–500.*

**The occurrence of biliary stricutures in allografts following liver transplantation correlates with the duration of preservation time. The correlation between preservation time and biliary strictures suggests that anoxic or reperfusion injury of the bile duct epithelium causes stricture formation. However, the relative susceptibility of bile duct cells to anoxic or reoxygenation injury is unknown. Our aims were to determine the vulnerability of rat liver bile duct cells to anoxic and reoxygenation injury and to compare the results with hepatocytes. During anoxia, bile duct epithelial cells were significantly more resistant to cell killing than hepatocytes. Rates of cellular proteolysis were also 2.5-fold lower in bile duct cells than in hepatocytes during anoxia. In contrast to anoxia, reoxygenation of anoxic cells increased cell killing of bile duct cells but improved viability of hepatocytes. The rate of toxic oxygen species formation by bile duct cells was 5-fold greater than in hepatocytes during reoxygenation. In addition, basal levels of glutathione are lower in bile duct cells than in hepatocytes. These data suggests that bile duct cells are more susceptible to reoxygenation injury than to anoxia. These studies support the hypothesis that reoxygenation injury during liver preservation leads to bile duct injury during liver transplantation.**

KEY WORDS: Bile duct strictures   liver transplant   liver storage time.

### PAPER DISCUSSION

Limitations in organ preservation still constitute a major problem in organ transplantation. The introduction of the University of Wisconsin (UW) solution in the late 1980's was seen as one of the major advances in liver transplantation[1,2]. With the UW solution liver grafts could be preserved for significantly longer periods. However, although the experimental data suggested that liver grafts could be preserved for beyond 24 hours, this initial optimism was not confirmed by the early clinical results. The large European multicenter study found that liver grafts preserved for longer than 12 hours were associated with poorer initial graft function, a greater incidence of primary non-function, more hepatocyte necrosis and poorer longterm outcome.

Preservation injury of the liver allograft consists of both an ischaemic injury and a reperfusion injury. Clearly the UW solution protects the liver against the ischaemic component of the preservation injury. However, reperfusion injury is being cited increasingly as a cause of primary nonfunction or poor early graft function and specific strategies directed at this component are now being introduced[3,4].

Non-anastomotic biliary strictures involving the biliary tree of the liver allograft have been identified with increasing frequency[5]. The occurrence of these bile duct strictures correlates with the duration of cold ischaemic storage time. It has been suggested that both an anoxic and reperfusion injury of the bile duct epithelium are involved in the pathogenesis of bile duct stricture formation. Since different mechanisms are involved in these two types of injury, it seems important to determine the relative role of each type of injury in the pathogenesis of bile duct strictures. In the above study, Noack *et al.* have used novel techniques to compare the vulnerability of bile duct epithelial cells to anoxic and reperfusion injuries.

Recent developments in the technology of isolating and separating bile duct epithelial cells and hepatocytes have made such studies possible[6]. In this study suspensions of bile duct cells and hepatocytes were subjected to variable periods of anoxia, followed by exposure to oxygen. Cell viability and degree of proteolysis were determined after anoxia and after reoxygenation. After anoxia, the cell viability was significantly greater and the magnitude of proteolysis significantly less for bile duct epithelial cells than for hepatocytes. Interestingly, reoxygenation of the bile duct epithelial cells resulted in increased cell death as compared with anoxia. In contrast reoxygenation of the hepatocytes was associated with increased survival. Following reoxygenation, the rates of formation of toxic oxygen species were significantly greater in bile duct cells as compared with hepatocytes. Bile duct cells were also shown to contain less glutathione. Thus bile duct epithelial cells appear to be more resistant to anoxia and more susceptible to the reoxygenation injury as compared with hepatocytes.

The authors acknowledge that the formation of bile duct strictures following liver transplantation may be the result of an injury to the microvasculature of the bile duct arteriolar plexus which would cause an ischaemic bile duct injury. Vascular endothelial cells, like bile duct cells, are susceptible to reoxygenation injury and resistant to anoxia. Thus, although reoxygenation appears to be the mechanism of bile duct injury, the precise principle target cell could be either the bile duct epithelial cell or the vascular endothelial cell.

The authors also acknowledge that these findings are limited both by the *in vitro* nature of the experiments and by the use of cells derived from rat livers since susceptibility to reperfusion injury does differ between species. For example, human hepatocytes are more resistant to anoxia-reoxygenation injury than rat hepatocytes[7]. Thus extrapolation to the *in vivo* situation is difficult and is compounded by the multiple variables present in the clinical setting.

Despite these limitations, these findings are both interesting and have several important implications. Firstly, these studies provide important information about the pathophysiology of bile duct injury during liver transplantation. The greater vulnerability of bile duct cells to reoxygenation injury and relative resistance to anoxia has important therapeutic implications. Further improvements in preservation solutions would have to address both the problems of anoxia as well as the reoxygenation; in fact, free radical scavengers are an important component of the new preservation solutions. "Wash-out" solutions, such as the Carolina Rinse solution for liver transplantation, also contain free radical scavengers[8]. However, the use of free radical scavengers has not been optimised and it may be necessary to administer these scavengers several hours before the procurement of organs for transplantation or to treat the recipient for several hours before transplantation.

The new "wash-out" solutions in liver transplantation have been developed to minimise the reperfusion injury[9]. Carolina rinse solution has been shown to minimise the degree of liver injury after liver transplantation. Recent studies have shown that flushing the liver with portal blood prior to revascularisation protects against reperfusion, and decreases the amount of liver injury and improves early graft function[10]. It would be interesting to see if these new strategies directed at the reperfusion injury influence the incidence of non-anastomotic bile duct strictures.

The finding of an increased susceptibility of bile duct cells to reperfusion injury is compatible with the report that Carolina Rinse solution, designed to minimise reperfusion injury, was more effective in improving markers of cholestatic injury than in decreasing markers of hepatocellular necrosis[8]. In other words the bile ducts appear to bear the brunt of the reperfusion injury.

The experiments described by Noack *et al.* can obviously be extended to other transplantable solid organs. For example, the relative susceptibility of glomerular cells and renal tubular cells to either anoxia or reoxygenation would be of interest. Other organs affected by disease processes in which the reperfusion injury has been implicated can also be studied using the techniques described above.

### REFERENCES

1. Jamieson, N.V., Lindell, S., Sundberg, R., Southard, J.H. and Belzer, F.O. (1988) An analysis of the components of UW solution using the isolated perfused rabbit liver. *Transplantation*, **46**, 512–516.
2. Kalayoglu, M., Sollinger, H.W. and Stratta, R.J., *et al.* (1988) Extended preservation of the liver for clinical transplantation. *Lancet*, **1**, 617–619.
3. Thurman, R.G., Marzi I., Seitz , G., Thies, J., Lemasters, J.J. and Zimmerman, F.A. (1988) Hepatic reperfusion injury following orthotopic liver transplantation in the rat. *Transplantation*, **46**, 502.
4. Petrowsky, H., Dippe, B. and Beck. P., *et al.* (1995) Do oxygen radicals play a role in primary dysfunction transplanted livers following preservation in University of Wisconsin solution? *Transplant. Proc.*, **27**, 729–731.
5. Sanchez-Urdazpal, L., Gores, G.J. and Lemasters, J.J., *et al.* (1993) Carolina Rinse solution decreases liver injury during clinical liver transplantation. *Transplant. Proc.*, **25**, 1574–1575.
6. Ishii, M., Vroman, B. and LaRusso, N.F. (1989) Isolation and morphologic characterization of bile duct epithelial cells from normal rat liver. *Gastroenterology* **97**, 1236.
7. Caraceni, P., Gasbarrini, A. and Rya, H.S., *et al.* (1994) Human hepatocytes differ from rat hepatocytes in their sensitivity to anoxia-reperfusion injury. *Transplant. Proc.*, **26**, 3307–3308.
8. Emre, S., Schwartz, M.E. and Mor, E., *et al.* (1994) Obviation of prereperfusion rinsing and decrease in preservation/reperfusion injury in liver transplantation by portal blood flushing. *Transplanation*, **57**, 799–803.
9. Sanchez-Urdazpal, L., Gores, G.J. and Ward, E.M., *et al.* (1992) Ischaemic-type biliary complications after orthotopic liver transplantation. *Hepatology*, **16**, 49–53.
10. Menegaux, F., Egawa, H., Keeffe, E.B., So, S.K., Concepcion, W., Collins, G.M. and Esquivel, C.O. (1993) Comparative effects of blood, Colloid, and Ringer's lactate terminal allograft rinse on the results of orthotopic liver transplantation. *Transplant. Proc.*, **25**, 3196–3198.

Professor D. Kahn
Head: Organ Transplant Unit
Department of Surgery
University of Cape Town Medical School
Observatory 7925
Cape Town
South Africa

### RESECTION MARGINS FOR COLORECTAL METASTASES TO THE LIVER: DO THEY MAKE A DIFFERENCE?

### ABSTRACT

*Yamamoto, J., Sugihara, K., Kosuge, T., Takayama, T., Shimada, K., Yamasaki, S., Sakamoto, M. and Hirohashi, S. (1995) Pathologic support for limited hepatectomy in the treatment of liver metastases from colorectal cancer. Annals of Surgery, 221*: 74–78

### Objective

**The authors determined an appropriate surgical treatment for liver metastases from colorectal cancers. Clinicopathologic features of metastatic lesions of colorectal cancers were studied.**

### Summary Background Data

**Major hepatic resection is the usual procedure for treatment of hepatic metastases from colorectal cancers.**

